# Intra and inter: Alterations in functional brain resting-state networks in patients with functional constipation

**DOI:** 10.3389/fnins.2022.957620

**Published:** 2022-07-22

**Authors:** Dan Zhang, Zai-Long Zhou, Ting Xing, Mei-Yu Zhou, Ye-Ming Wan, Shu-Chen Chang, Ya-Li Wang, Hai-Hua Qian

**Affiliations:** ^1^Department of Anorectal Surgery, The Affiliated Hospital of Nanjing University of Chinese Medicine, Nanjing, China; ^2^No. 1 Clinical Medical College, Nanjing University of Chinese Medicine, Nanjing, China

**Keywords:** functional constipation, resting-state networks, independent component analysis, functional network connectivity, brain plasticity

## Abstract

**Background:**

Functional constipation (FCon), is a symptom-based functional gastrointestinal disorder without an organic etiology and altering brain structure and function. However, previous studies mainly focused on isolated brain regions involved in brain plasticity. Therefore, little is known about the altered large-scale interaction of brain networks in FCon.

**Methods:**

For this study, we recruited 20 patients with FCon and 20 healthy controls. We used group independent component analysis to identify resting-state networks (RSNs) and documented intra- and inter-network alterations in the RSNs of the patients with FCon.

**Results:**

We found 14 independent RSNs. Differences in the intra-networks included decreased activities in the bilateral caudate of RSN 3 (strongly related to emotional and autonomic processes) and decreased activities in the left precuneus of RSN 10 (default mode network). Notably, the patients with FCon exhibited significantly decreased interactive connectivity between RSNs, mostly involving the connections to the visual perception network (RSN 7–9).

**Conclusion:**

Compared with healthy controls, patients with FCon had extensive brain plastic changes within and across related RSNs. Furthermore, the macroscopic brain alterations in FCon were associated with interoceptive abilities, emotion processing, and sensorimotor control. These insights could therefore lead to the development of new treatment strategies for FCon.

## Introduction

Functional constipation (FCon), a symptom-based functional gastrointestinal disorder without an organic etiology, is characterized by difficulty in defecation, reduced stool frequency, and abdominal distension and pain (Mugie et al., 2011). The efficacy of drug-based and non-drug therapies (such as lifestyle and pelvic floor interventions) remains far from satisfactory, and the therapy cycle is long (Koppen et al., 2015; Rao et al., 2016).

With the development of modern neurogastroenterology and the in-depth study of the structure and function of the enteric nervous system, the links at different levels between the gastrointestinal system and the central nervous system have been thoroughly investigated (Jones et al., 2006; Heiss and Olofsson, 2019; Barrio et al., 2022). Moreover, neuroimaging is a recent convenient tool for studying the brain plastic changes derived from FCon. Brain mechanism researchers commonly use functional MRI (fMRI), for example to observe the brain structural and functional changes in FCon patients. Several recent studies have reported that FCon could alter brain structure and functions such as somatic and sensory processing, motor control, self-referential processing, and emotional process modulation (Zhu et al., 2016; Hu et al., 2020; Duan et al., 2021; Li et al., 2021; Liu et al., 2021; Zhang Z. et al., 2021). Moreover, the brain plastic changes might be closely associated with constipation symptoms and emotional status. However, previous studies mostly focused on isolated brain regions involved in brain plasticity. Moreover, behavior-related brain function depends on the functional integration of neural networks. A previous study employed graph theory to investigate the large-scale characteristics of the brain functional networks and indicated that FCon was related to reduced functional connectivity and abnormalities in the thalamocortical networks (Liu et al., 2021).

Neuroimaging studies have revealed that the brain is organized into spatially segregated and functionally integrated intrinsic connectivity networks (Laird et al., 2011). For example, the default mode network, which mainly includes the medial prefrontal cortex and the posterior cingulate cortex, is a task-negative network that is active during periods of inactivity (Raichle and Snyder, 2007). Independent component analysis (ICA) is a powerful and widely used data-driven method for detecting independent patterns in multivariate information (Fox and Raichle, 2007; Fang et al., 2021). Meanwhile, fMRI studies are also suitable for decomposing resting-state data into potential spatially segregated intrinsic connectivity networks, called resting-state networks (RSNs). RSN identification and functional connectivity analysis have provided an approach to investigating the macroscopic spatio-temporal organization of the brain (Lin et al., 2017; Xing et al., 2020; Zhang H. et al., 2021).

Herein, we used group independent component analysis (GICA) to identify RSNs. Next, we investigated intra- and inter-network alterations in the RSNs of FCon patients. This study is the first to explore the macroscopic spatio-temporal alterations within and across the related functional RSNs in patients with FCon.

## Materials and methods

### Participants

A total of 20 FCon patients and 20 healthy controls were recruited at the Affiliated Hospital of Nanjing University of Chinese Medicine (Jiangsu Province Hospital of Chinese Medicine). Two experienced gastroenterologists from the Affiliated Hospital of Nanjing University of Chinese Medicine (Jiangsu Province Hospital of Chinese Medicine) performed FCon diagnosis using the Rome IV criteria (Drossman, 2016). We also calculated the Constipation Scoring System (CSS) of the FCon patients. The CSS score quantifies constipation on a scale of 0–30 points, with a higher score indicating a worse constipation. We recruited FCon patients with no other medical or psychological disorders. Besides, the patients with the following conditions were excluded from our study: (1) redundant sigmoid colon/congenital giant colon/pelvic floor muscle relaxation; (2) constipation after childbirth; (3) current medications affecting brain function; and (4) contraindications to fMRI. In addition, we recruited age- and gender-matched healthy controls through advertisements placed in the local community. We also surveyed the FCon patients and healthy controls using the ZUNG self-rating depression scale (SDS) and ZUNG self-rating anxiety scale (SAS; Zung, 1971, 1965). To ensure the absence of anxiety or depression, we excluded participants with SAS and SDS total scores below 50. The Ethics Committee of the Affiliated Hospital of Nanjing University of Chinese Medicine (Jiangsu Province Hospital of Chinese Medicine) reviewed and approved this study involving human participants (2021-NL-044-02). The study protocol has been approved by the local research ethics committee and registered with the Chinese Clinical Trial Registry (ChiCTR2100048671). All the participants provided their written informed consent to participate in this study.

### Resting-state functional MRI data acquisition and preprocessing

We performed resting-state fMRI scans of the whole brain on the participants using a GE 3T MRI scanner (SIGNA Architect). Participants were instructed to lie still and had their heads immobilized with foam pads. The gradient echo-planar imaging sequence was used for scanning, and the scanning parameters were as follows: slice number = 49, TR = 2 s, matrix size = 128 × 128, FOV = 240 mm × 240 mm, flip angle = 90°, slice thickness = 3 mm, number of average = 2.

Resting-state fMRI data were preprocessed using the Statistical Parametric Mapping 8 toolbox^1^ implemented in the MATLAB 2014a platform. Briefly, the main steps were as follows: (1) removing the first 10 TRs data for the signal equilibrium; (2) slice-timing correction; (3) correction for head motion (the head movements were all < 2.5 mm or 2.5 degrees in any direction); (4) spatial normalization to the standard Montreal Neurological Institute space; (5) spatial smoothing with a 6-mm Gaussian kernel; and (6) temporal bandpass filtering (0.01–0.1 Hz) to decrease the low-frequency drift.

### Independent component analysis and resting-state networks identification

Following data preprocessing, we performed ICA—a data-driven analysis method (Calhoun et al., 2001)—to identify the resting-state independent components (ICs) of the FCon patients and healthy controls using GICA of fMRI Toolbox^2^.

We applied a concatenation approach plus back-reconstruction for the GICA. First, we reduced the dimensionality of the images using principal component analysis, then temporally concatenated the data and decomposed them into 39 components using the information-maximization (infomax) algorithm. Then, we estimated the ICs number according to the minimum description length criteria (Jafri et al., 2008). To assess the robustness, we applied 100 repetitions of the infomax ICA algorithm in ICASSO. Next, we reconstructed the spatial patterns and time courses of the group-level ICs for each subject. We then performed *z*-transformations of the ICA-determined maps. Finally, we identified 14 ICs-of-interest by spatial sorting and visual inspection according to a previous ICA analysis study (Laird et al., 2011).

### Analysis of the intra-network alterations within resting-state networks

We performed a one-sample *t*-test in SPM8 for each selected IC to get a group-level RSN spatial map for all subjects [*p* < 0.05 after false discovery rate (FDR) correction]. We also binarized the significant clusters to define them as mask. Subsequently, we investigated the intra-connectivity differences in each RSN by comparing two groups using two-sample *t*-tests (*p* < 0.05 after FDR correction) within the binary mask of each selected IC.

### Analysis of the inter-network alterations between resting-state networks

Next, we employed the functional network connectivity (FNC) toolbox^3^ to investigate the possible interaction between RSNs using a constrained maximal time-lagged correlation method reflecting the temporal domain interactions.

Based on the ICA algorithm, the source signals of brain regions within one component are synchronous. Thus, we extracted the time courses of each component and further measured the temporal synchronous degree between RSNs by Pearson correlation. We conducted one-sample *t*-tests to examine the temporal interactions between any pair of ICs for each group (*p* < 0.05 after FDR correction). Next, we performed two-sample *t*-tests for group comparison of all possible statistically significant correlation combinations (*p* < 0.05 after FDR correction).

## Results

### Demographic characteristics

The patient and control groups had similar age ranges and gender partitions. Although no participants were diagnosed with depression or anxiety (with SAS/SDS scores < 50), the FCon patients had significantly higher SAS (*p* = 0.0088) and SDS (*p* = 0.0001) scores than the healthy controls. The FCon patients’ average constipation duration was 7.3 ± 3.70 months and the CSS score was 13.50 ± 3.66 (Table 1).

**TABLE 1:** Demographic and clinical information of the participants.

	FCon patients	Healthy controls	*p*-value
Age (years)	41.41 ± 13.02	39.25 ± 11.44	0.44*^a^*
Gender (female/male)	7/13	8/12	0.74*^b^*
SDS	40.45 ± 7.36	34.86 ± 3.82	0.0088*^a^*
SAS	39.91 ± 5.50	38.81 ± 11.33	0.0001*^a^*
Duration of FCon (months)	7.30 ± 3.70	–	–
Constipation Scoring System scores	13.50 ± 3.66	–	–

FCon, functional constipation; RSN, resting-state networks. ^*a*^Two sample t-tests. ^*b*^Chi-square test.

### Resting-state networks identification

**FIGURE 1:**
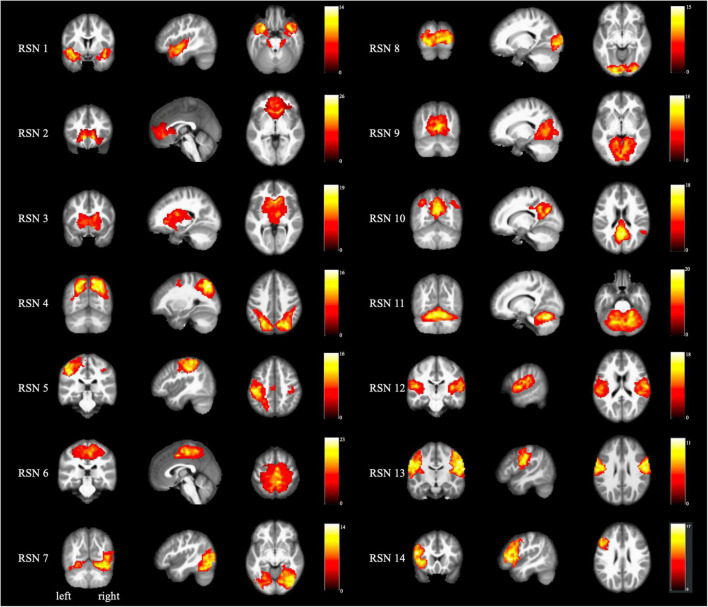
Spatial maps for each RSN. Each RSN map was obtained using one-sample *t*-test across all individual independent components (*p* < 0.05 after FDR correction). The color bar indicates the *t*-value of the one-sample *t*-test. RSN, resting-state networks.

We identified 14 ICs by spatial sorting and visual inspection according to a previous ICA analysis study (Laird et al., 2011), and we specified each RSN map using one-sample *t*-tests across all individual IC maps. We describe each of these briefly below, as Laird et al. (2011) previously reported, and Figure 1 shows the cluster location of each IC.

RSN 1 (limbic and medial-temporal regions) was strongly associated with interoceptive processing caused by air-hunger, and with the olfactory and gustatory responses, albeit more weakly.

RSN 2 (subgenual anterior cingulate cortex and orbitofrontal cortex) was relevant to olfaction, gustation, and emotion and strongly associated with the reward and thirst tasks (Heatherton, 2011).

RSN 3 (bilateral basal ganglia and thalamus) was strongly related to reward tasks and interoceptive processing caused by hunger, thirst, as well as anxiety and olfaction (Herrero et al., 2002).

RSN 4 (middle frontal and superior parietal areas) was related to visuospatial processing and reasoning (Dosenbach et al., 2007).

RSN 5 (ventral precentral gyri, central sulci, postcentral gyri, superior, and inferior cerebellum) was linked to action and somesthesis relevant to hand movements and tasks (Smith et al., 2009).

RSN 6 (superior parietal lobule) was anticorrelated with some cognitive and emotional processing (Scheperjans et al., 2005).

RSN 7 (middle and inferior temporal areas) was related to visual perception tasks, including viewing complex, usually emotional stimuli (such as visual food stimuli; Masterson et al., 2019).

RSN 8 and 9 (lateral and medial posterior occipital areas) were also associated with visual perception (Ishioka et al., 2021). RSN 11 was strongly weighted toward higher-level visual processing such as orthography and covert reading. Meanwhile, RSN 12 was mainly associated with simple visual stimuli such as flashing checkerboards (White et al., 2011).

RSN 10 (medial prefrontal and posterior cingulate/precuneus areas), also known as the default mode network, was closely associated with theory of mind and social cognition tasks (Biswal et al., 2010).

RSN 11 (cerebellum) was commonly related to action and somesthesis, as well as several sensorimotor, autonomic, and cognitive functions (Stoodley, 2016).

RSN 12 (transverse temporal gyri) was associated with audition, music, and speech (Fishman et al., 2001).

RSN 13 (dorsal precentral gyri, central sulci, postcentral gyri, and superior and inferior cerebellum) included primary sensorimotor cortices for the mouth. It was linked to action and somesthesis involving speech, chewing, or swallowing, and tongue motor function (Ekstrand et al., 2019).

RSN 14 (left-lateralized frontoparietal areas) was closely associated with a distributed range of semantic, phonologic, and orthographic language tasks, as well as working and explicit memory tasks (Yang and Zevin, 2014).

### The intra-network difference between functional constipation patients and healthy controls in identified resting-state networks

**FIGURE 2:**
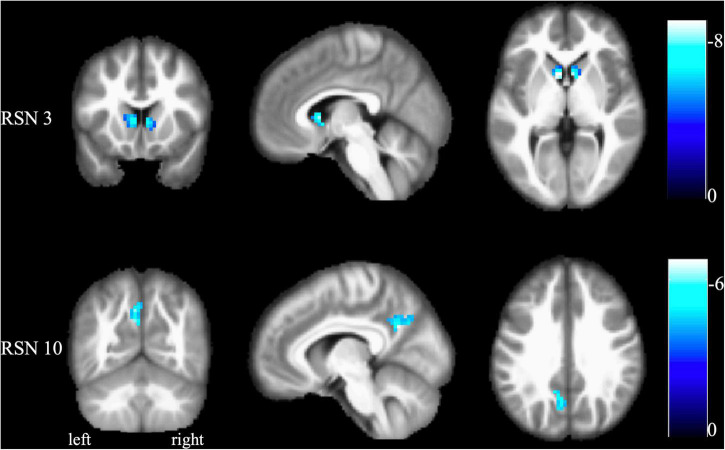
The results of the intranetwork difference between FCon patients and healthy controls. Altered functional activities were found in RSN 3 (bilateral basal ganglia and thalamus) and RSN 10 (default mode network). No difference was found in other RSNs. The color bar indicates the *t*-value of the two-sample *t*-test between FCon patients and healthy controls. A negative *t*-value means significantly lower functional activity in the FCon patients. FCon, functional constipation; RSN, resting-state networks.

Figure 2 and Table 2 present the intra-network differences. FCon patients had significantly lower bilateral caudate nucleus activities in the RSN 3 than the healthy controls. They also had significantly decreased activity in the left precuneus (RSN 10). We found no differences in other RSNs.

**TABLE 2:** The intranetwork difference in identified RSNs between FCon patients and healthy controls.

Brain region	Cluster size	MNI coordinates			*t*-value
		*x*	*y*	*z*	
*RSN 3*					
Left caudate	88	−6	12	3	−8.58
Right caudate	65	15	0	18	−7.89
*RSN 10*					
Left precuneus	86	−9	−60	30	−6.74

FCon, functional constipation; RSN, resting-state networks.

### The interactive alterations between the resting-state networks

**FIGURE 3:**
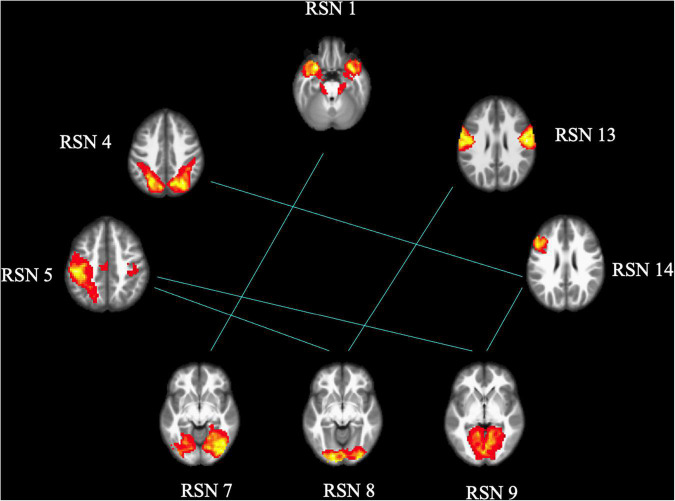
FCon-related differences in brain functional network connectivity between RSNs. The blue line between relative RSNs means significantly lower connectivity strength in FCon patients. FCon, functional constipation; RSN, resting-state networks.

**FIGURE 4:**
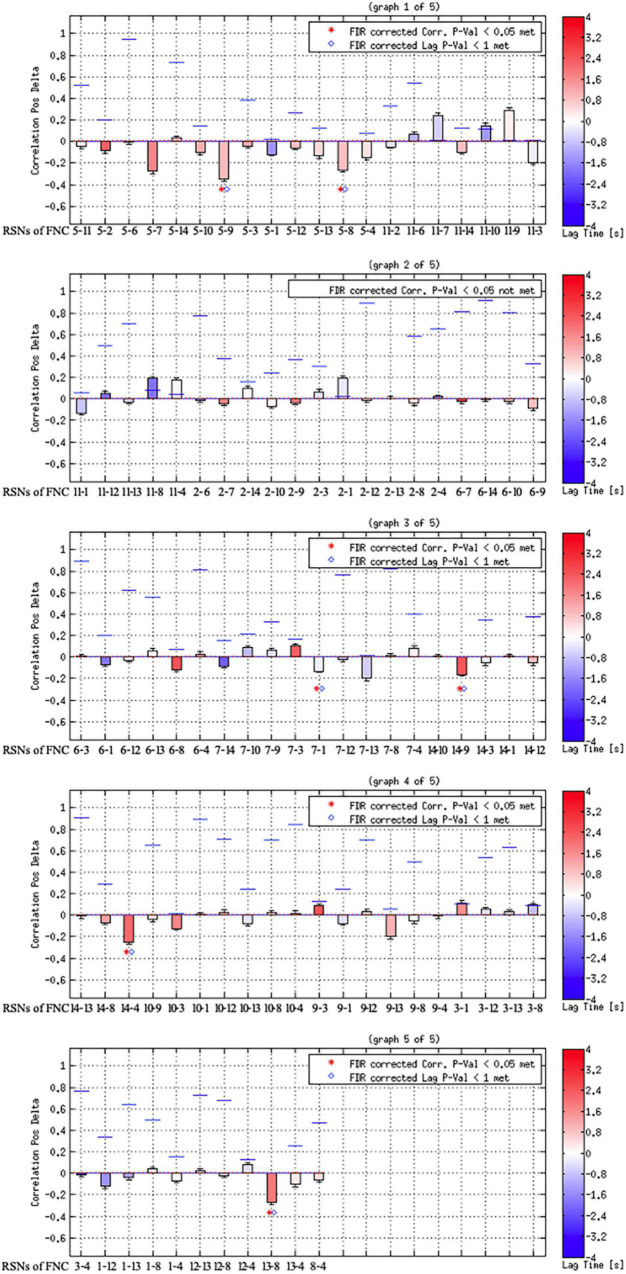
Results of component combination between each pair of the identified RSNs. The blue lines show the correlation of *p*-value; the red dotted line shows the user *p*-value threshold.

As shown in Figures 3, 4, FCon patients showed significantly lower connectivity between RSN 1 and RSN 7, RSN 5, and RSN 9, RSN 5 and RSN 8, RSN 4 and RSN 14, RSN 5 and RSN 9, RSN 9 and RSN 14, and RSN 8 and RSN 13 than healthy controls. Notably, there was no significantly increased connectivity in FCon patients.

## Discussion

This study investigated the difference in the intra-network and interactive alterations of RSNs between FCon patients and healthy controls using ICA and FNC algorithms. Differences in the intra-network showed decreased activities in the bilateral caudate nucleus of RSN 3 (strongly related to emotional and autonomic processes) and decreased activities in the left precuneus of RSN 10 (default mode network). Notably, the FCon patients exhibited significantly decreased interactive connectivity between RSNs, primarily including the connections to the visual perception network (RSNs 7, 8, and 9). Overall, FCon patients had extensive brain plastic changes within and across related RSNs compared with healthy controls.

Using the ICA approach, we identified 14 RSNs. RSNs 1–3 were strongly linked to emotional and autonomic processes involving interoceptive processes, such as hunger and thirst. RSNs 4–6 were closely related to motor and visuospatial integration, coordination, and execution. RSNs 7–9 were associated with visual perception. Finally, RSNs 10–14 were divergent networks known as the default mode network (10), cerebellum network (11), auditory network (12), motor and sensory cortices for the mouth (13), and left frontoparietal regions (14).

The intra-network activities analysis revealed that FCon patients had lower activities in the bilateral caudate nucleus, a basal ganglia brain region required for executive functions (Macfarlane et al., 2013). Furthermore, the caudate nucleus has consistently been linked to reward processing (O’Doherty, 2004; Knutson and Cooper, 2005). Studies of irritable bowel syndrome (IBS) specific brain alterations indicated that the basal ganglia played a role in pain processing by undergoing microstructural and functional reorganization (Song et al., 2006; Tillisch et al., 2011; Ellingson et al., 2013). Furthermore, deep brain stimulation of the caudate nucleus effectively relieved both psychiatric and gastrointestinal symptoms in a patient with IBS (Langguth et al., 2015). FCon, like IBS, may be linked to abnormal gastrointestinal sensorimotor and emotional processing.

Additionally, the FCon patients showed lower activities in the left precuneus of RSN 10 (default mode network), which is crucial in self-referential processing and mood control (Raichle and Snyder, 2007). Buvanendran (2010) found that the precuneus was activated in patients with chronic pain and was linked to self-awareness; thus, it might be associated with these patients’ perception of their relationship with the outside world (Cavanna and Trimble, 2006). Besides, numerous studies have focused on the emotional problems of patients with FCon (Dykes et al., 2001; Chan et al., 2005; Duan et al., 2021; Li et al., 2021). They reported that FCon was related to increased psychosocial distress. Furthermore, neuroimaging studies found that FCon patients with anxiety or depression showed significantly different basal brain activities and connectivity in the emotional-arousal network and thalamus. Similarly, FCon patients with anxiety or depression had significantly different functional connectivity within and between salience and sensorimotor networks (Duan et al., 2021; Li et al., 2021). Overall, the physiological abnormalities in the gastrointestinal system are apparently associated with psychological factors.

Another key finding of this study was that FCon only significantly decreased the interactive connectivity between RSNs, mostly involving the connections to the visual perception network (RSN 7–9). The other RSNs of the connection included those associated with motor control and execution (RSNs 4 and 5), hunger-satiety perception (RSN 1), motor and sensory cortices for the mouth (RSN 13), and working and explicit memory (RSN 14). These connections between RSNs are primarily related to interoception, which is crucial to emotional processes. Several psychiatric disorders, such as eating disorders (Perry et al., 2021) and depression (Eggart et al., 2021; Hielscher and Zopf, 2021), impair these processes. Regarding somatic diseases such as IBD, previous studies have focused on interoceptive abilities and their role in emotional processing (Longarzo et al., 2017; Atanasova et al., 2021). In light of our findings, future research on FCon should focus on interoceptive abilities, emotion processing, and sensorimotor control.

## Limitations

First, this study had a relatively small sample size, limiting our results’ generalizability and statistical power. Second, we excluded patients with FCon accompanied by anxiety or depression and therefore did not compare them with FCon patients without anxiety or depression. Although the patients had only slightly higher SAS and SDS scores than the healthy controls, none of them met the diagnostic criteria of anxiety or depression. Finally, the lack of interoceptive abilities assessment in patients with FCon is a limitation, especially since the decreased connections were found between RSNs mostly associated with interoception. Future work should, therefore, further investigate the association between brain functional and structural changes and interoceptive abilities.

## Conclusion

In this study, we clarified the intra- and inter-networks alterations in patients with FCon. Overall, our results support the notion that the macroscopic brain alterations in FCon are associated with interoceptive abilities, emotion processing, and sensorimotor control. These insights could lead to new treatment strategies for FCon.

## Data availability statement

The raw data supporting the conclusions of this article will be made available by the authors, without undue reservation.

## Ethics statement

The studies involving human participants were reviewed and approved by the Ethics Committee of The Affiliated Hospital of Nanjing University of Chinese Medicine (Jiangsu Province Hospital of Chinese Medicine). The patients/participants provided their written informed consent to participate in this study.

## Author contributions

DZ and TX drafted the initial manuscript. DZ, Z-LZ, and M-YZ collected and preprocessed the functional MRI data. Y-MW and H-HQ designed experiments and analyzed the results. Y-LW, S-CC, and H-HQ revised the manuscript. All authors contributed to the article and approved the submitted version.

## Conflict of interest

The authors declare that the research was conducted in the absence of any commercial or financial relationships that could be construed as a potential conflict of interest.

## Publisher’s note

All claims expressed in this article are solely those of the authors and do not necessarily represent those of their affiliated organizations, or those of the publisher, the editors and the reviewers. Any product that may be evaluated in this article, or claim that may be made by its manufacturer, is not guaranteed or endorsed by the publisher.

## Footnotes

1
http://www.fil.ion.ucl.ac.uk/spm/

2 version 4.0, http://icatb.sourceforge.net

3
http://mialab.mrn.org/software/#fnc

## References

[B1] Atanasova K. Lotter T. Reindl W. Lis S. (2021). Multidimensional assessment of interoceptive abilities, emotion processing and the role of early life stress in inflammatory bowel diseases. *Front. Psychiatry* 12:680878. 10.3389/fpsyt.2021.680878 34248716 PMC8264143

[B2] Barrio C. Arias-Sánchez S. Martín-Monzón I. (2022). The gut microbiota-brain axis, psychobiotics and its influence on brain and behaviour: a systematic review. *Psychoneuroendocrinology* 137:105640. 34942539 10.1016/j.psyneuen.2021.105640

[B3] Biswal B. B. Mennes M. Zuo X.-N. Gohel S. Kelly C. Smith S. M. (2010). Toward discovery science of human brain function. *Proc. Natl. Acad. Sci. U.S.A.* 107 4734–4739. 20176931 10.1073/pnas.0911855107 PMC2842060

[B4] Buvanendran A. (2010). Brain activity associated with chroniccancer pain. *Pain Physician* 13 E342–E342. 20859325

[B5] Calhoun V. D. Adali T. Pearlson G. D. Pekar J. J. (2001). A method for making group inferences from functional MRI data using independent component analysis. *Hum. Brain Mapp.* 14 140–151. 11559959 10.1002/hbm.1048 PMC6871952

[B6] Cavanna A. E. Trimble M. R. (2006). The precuneus: a review of its functional anatomy and behavioural correlates. *Brain* 129 564–583. 16399806 10.1093/brain/awl004

[B7] Chan A. O. Cheng C. Hui W. M. Hu W. H. Wong N. Y. Lam K. (2005). Differing coping mechanisms, stress level and anorectal physiology in patients with functional constipation. *World J. Gastroenterol.* 11 5362–5366. 10.3748/wjg.v11.i34.5362 16149147 PMC4622810

[B8] Dosenbach N. U. F. Fair D. A. Miezin F. M. Cohen A. L. Wenger K. K. Dosenbach R. A. T. (2007). Distinct brain networks for adaptive and stable task control in humans. *Proc. Natl. Acad. Sci. U.S.A.* 104 11073–11078. 17576922 10.1073/pnas.0704320104 PMC1904171

[B9] Drossman D. A. (2016). Functional gastrointestinal disorders: history, pathophysiology, clinical features, and Rome IV. *Gastroenterology* 150 1262–1279.e2. 10.1053/j.gastro.2016.02.032 27144617

[B10] Duan S. Liu L. Li G. Wang J. Hu Y. Zhang W. (2021). Altered functional connectivity within and between salience and sensorimotor networks in patients with functional constipation. *Front. Neurosci.* 15:628880. 10.3389/fnins.2021.628880 33776637 PMC7991789

[B11] Dykes S. Smilgin-Humphreys S. Bass C. (2001). Chronic idiopathic constipation: a psychological enquiry. *Eur. J. Gastroenterol. Hepatol.* 13 39–44. 10.1097/00042737-200101000-00007 11204807

[B12] Eggart M. Todd J. Valdés-Stauber J. (2021). Validation of the multidimensional assessment of interoceptive awareness (MAIA-2) questionnaire in hospitalized patients with major depressive disorder. *PLoS One* 16:e0253913. 10.1371/journal.pone.0253913 34170963 PMC8232409

[B13] Ekstrand C. Neudorf J. Gould L. Mickleborough M. Borowsky R. (2019). Where words and space collide: the overlapping neural activation of lexical and sublexical reading with voluntary and reflexive spatial attention. *Brain Res.* 1706 1–12. 10.1016/j.brainres.2018.10.022 30347218

[B14] Ellingson B. M. Mayer E. Harris R. J. Ashe-McNally C. Naliboff B. D. Labus J. S. (2013). Diffusion tensor imaging (DTI) detects microstructural reorganization in the brain associated with chronic irritable bowel syndrome (IBS). *Pain* 154 1528–1541. 10.1016/j.pain.2013.04.010 23721972 PMC3758125

[B15] Fang X. T. Toyonaga T. Hillmer A. T. Matuskey D. Holmes S. E. Radhakrishnan R. (2021). Identifying brain networks in synaptic density PET (11C-UCB-J) with independent component analysis. *NeuroImage* 237:118167. 10.1016/j.neuroimage.2021.118167 34000404 PMC8452380

[B16] Fishman Y. I. Volkov I. O. Noh M. D. Garell P. C. Bakken H. Arezzo J. C. (2001). Consonance and dissonance of musical chords: neural correlates in auditory cortex of monkeys and humans. *J. Neurophysiol.* 86 2761–2788. 11731536 10.1152/jn.2001.86.6.2761

[B17] Fox M. D. Raichle M. E. (2007). Spontaneous fluctuations in brain activity observed with functional magnetic resonance imaging. *Nat. Rev. Neurosci.* 8 700–711. 17704812 10.1038/nrn2201

[B18] Heatherton T. F. (2011). Neuroscience of self and self-regulation. *Annu. Rev. Psychol.* 62 363–390. 21126181 10.1146/annurev.psych.121208.131616 PMC3056504

[B19] Heiss C. N. Olofsson L. E. (2019). The role of the gut microbiota in development, function and disorders of the central nervous system and the enteric nervous system. *J. Neuroendocrinol.* 31:e12684. 30614568 10.1111/jne.12684

[B20] Herrero M.-T. Barcia C. Navarro J. (2002). Functional anatomy of thalamus and basal ganglia. *Childs Nerv. Syst.* 18 386–404. 12192499 10.1007/s00381-002-0604-1

[B21] Hielscher E. Zopf R. (2021). Interoceptive abnormalities and suicidality: a systematic review. *Behav. Ther.* 52 1035–1054. 34452660 10.1016/j.beth.2021.02.012

[B22] Hu C. Liu L. Liu L. Zhang J. Hu Y. Zhang W. (2020). Cortical morphometry alterations in brain regions involved in emotional, motor-control and self-referential processing in patients with functional constipation. *Brain Imaging Behav.* 14 1899–1907. 31218532 10.1007/s11682-019-00133-4

[B23] Ishioka T. Hirayama K. Hosokai Y. Takeda A. Suzuki K. Nishio Y. (2021). Impaired perception of illusory contours and cortical hypometabolism in patients with Parkinson’s disease. *Neuroimage Clin.* 32:102779. 10.1016/j.nicl.2021.102779 34418792 PMC8385116

[B24] Jafri M. J. Pearlson G. D. Stevens M. Calhoun V. D. (2008). A method for functional network connectivity among spatially independent resting-state components in schizophrenia. *Neuroimage* 39 1666–1681. 18082428 10.1016/j.neuroimage.2007.11.001 PMC3164840

[B25] Jones M. P. Dilley J. B. Drossman D. Crowell M. D. (2006). Brain–gut connections in functional GI disorders: anatomic and physiologic relationships. *Neurogastroenterol. Motil.* 18 91–103. 10.1111/j.1365-2982.2005.00730.x 16420287

[B26] Knutson B. Cooper J. C. (2005). Functional magnetic resonance imaging of reward prediction. *Curr. Opin. Neurol.* 18 411–417. 16003117 10.1097/01.wco.0000173463.24758.f6

[B27] Koppen I. J. N. Lammers L. A. Benninga M. A. Tabbers M. M. (2015). Management of functional constipation in children: therapy in practice. *Paediatr. Drugs* 17 349–360. 26259965 10.1007/s40272-015-0142-4 PMC4768242

[B28] Laird A. R. Fox P. M. Eickhoff S. B. Turner J. A. Ray K. L. McKay D. R. (2011). Behavioral interpretations of intrinsic connectivity networks. *J. Cogn. Neurosci.* 23 4022–4037. 21671731 10.1162/jocn_a_00077 PMC3690655

[B29] Langguth B. Sturm K. Wetter T. C. Lange M. Gabriels L. Mayer E. A. (2015). Deep brain stimulation for obsessive compulsive disorder reduces symptoms of irritable bowel syndrome in a single patient. *Clin. Gastroenterol. Hepatol.* 13 1371–1374.e3. 10.1016/j.cgh.2015.01.023 25638586 PMC4986991

[B30] Li G. Zhang W. Hu Y. Wang J. Li J. Jia Z. (2021). Distinct basal brain functional activity and connectivity in the emotional-arousal network and thalamus in patients with functional constipation associated with anxiety and/or depressive disorders. *Psychosom. Med.* 83 707–714. 34117157 10.1097/PSY.0000000000000958

[B31] Lin W. Wu H. Liu Y. Lv D. Yang L. (2017). A CCA and ICA-based mixture model for identifying major depression disorder. *IEEE Transac. Med. Imaging* 36 745–756. 10.1109/TMI.2016.2631001 27893387

[B32] Liu L. Hu C. Hu Y. Zhang W. Zhang Z. Ding Y. (2021). Abnormalities in the thalamo-cortical network in patients with functional constipation. *Brain Imaging Behav.* 15 630–642. 10.1007/s11682-020-00273-y 32314199

[B33] Longarzo M. Quarantelli M. Aiello M. Romano M. Del Prete A. Cimminiello C. (2017). The influence of interoceptive awareness on functional connectivity in patients with irritable bowel syndrome. *Brain Imaging Behav.* 11 1117–1128. 10.1007/s11682-016-9595-5 27704405

[B34] Macfarlane M. D. Looi J. C. L. Walterfang M. Spulber G. Velakoulis D. Crisby M. (2013). Executive dysfunction correlates with caudate nucleus atrophy in patients with white matter changes on MRI: a subset of LADIS. *Psychiatry Res. Neuroimaging* 214 16–23. 10.1016/j.pscychresns.2013.05.010 23916538

[B35] Masterson T. D. Bermudez M. A. Austen M. Lundquist E. Pearce A. L. Bruce A. S. (2019). Food commercials do not affect energy intake in a laboratory meal but do alter brain responses to visual food cues in children. *Appetite* 132 154–165. 30312738 10.1016/j.appet.2018.10.010 PMC7061687

[B36] Mugie S. M. Benninga M. A. Di Lorenzo C. (2011). Epidemiology of constipation in children and adults: a systematic review. *Best Pract. Res. Clin. Gastroenterol.* 25 3–18. 21382575 10.1016/j.bpg.2010.12.010

[B37] O’Doherty J. P. (2004). Reward representations and reward-related learning in the human brain: insights from neuroimaging. *Curr. Opin. Neurobiol.* 14 769–776. 15582382 10.1016/j.conb.2004.10.016

[B38] Perry T. R. Wierenga C. E. Kaye W. H. Brown T. A. (2021). Interoceptive awareness and suicidal ideation in a clinical eating disorder sample: the role of body trust. *Behav. Ther.* 52 1105–1113. 10.1016/j.beth.2020.12.001 34452665

[B39] Raichle M. E. Snyder A. Z. (2007). A default mode of brain function: a brief history of an evolving idea. *Neuroimage* 37 1083–1090. 10.1016/j.neuroimage.2007.02.041 17719799

[B40] Rao S. S. C. Rattanakovit K. Patcharatrakul T. (2016). Diagnosis and management of chronic constipation in adults. *Nat. Rev. Gastroenterol. Hepatol.* 13 295–305. 27033126 10.1038/nrgastro.2016.53

[B41] Scheperjans F. Grefkes C. Palomero-Gallagher N. Schleicher A. Zilles K. (2005). Subdivisions of human parietal area 5 revealed by quantitative receptor autoradiography: a parietal region between motor, somatosensory, and cingulate cortical areas. *Neuroimage* 25 975–992. 10.1016/j.neuroimage.2004.12.017 15808998

[B42] Smith S. M. Fox P. T. Miller K. L. Glahn D. C. Fox P. M. Mackay C. E. (2009). Correspondence of the brain’s functional architecture during activation and rest. *Proc. Natl. Acad. Sci. U.S.A.* 106 13040–13045. 19620724 10.1073/pnas.0905267106 PMC2722273

[B43] Song G. H. Venkatraman V. Ho K. Y. Chee M. W. L. Yeoh K. G. Wilder-Smith C. H. (2006). Cortical effects of anticipation and endogenous modulation of visceral pain assessed by functional brain MRI in irritable bowel syndrome patients and healthy controls. *Pain* 126 79–90. 10.1016/j.pain.2006.06.017 16846694

[B44] Stoodley C. J. (2016). The cerebellum and neurodevelopmental disorders. *Cerebellum* 15 34–37. 26298473 10.1007/s12311-015-0715-3 PMC4811332

[B45] Tillisch K. Mayer E. A. Labus J. S. (2011). Quantitative meta-analysis identifies brain regions activated during rectal distension in irritable bowel syndrome. *Gastroenterology* 140 91–100. 10.1053/j.gastro.2010.07.053 20696168 PMC3253553

[B46] White T. Moeller S. Schmidt M. Pardo J. V. Olman C. (2011). Evidence for intact local connectivity but disrupted regional function in the occipital lobe in children and adolescents with schizophrenia. *Hum. Brain Mapp.* 33 1803–1811. 10.1002/hbm.21321 21674696 PMC4194193

[B47] Xing X. Hua X. Zheng M. Ma Z. Huo B. Wu J. (2020). Intra and inter: alterations in functional brain resting−state networks after peripheral nerve injury. *Brain Behav.* 10:e01747. 10.1002/brb3.1747 32657022 PMC7507705

[B48] Yang J. Zevin J. (2014). The impact of task demand on visual word recognition. *Neuroscience* 272 102–115. 24814725 10.1016/j.neuroscience.2014.04.044

[B49] Zhang H. Zeng W. Deng J. Shi Y. Zhao L. Li Y. (2021). Brain relatively inert network: taking adult attention deficit hyperactivity disorder as an example. *Front. Neurosci.* 15:771947. 10.3389/fnins.2021.771947 34924940 PMC8678527

[B50] Zhang Z. Hu Y. Lv G. Wang J. He Y. Zhang L. (2021). Functional constipation is associated with alterations in thalamo-limbic/parietal structural connectivity. *Neurogastroenterol. Motil.* 33:e13992. 33073892 10.1111/nmo.13992

[B51] Zhu Q. Cai W. Zheng J. Li G. Meng Q. Liu Q. (2016). Distinct resting-state brain activity in patients with functional constipation. *Neurosci. Lett.* 632 141–146. 27569716 10.1016/j.neulet.2016.08.042

[B52] Zung W. W. K. (1965). A self-rating depression scale. *Arch. Gen. Psychiatry* 12:63. 14221692 10.1001/archpsyc.1965.01720310065008

[B53] Zung W. W. K. (1971). A rating instrument for anxiety disorders. *Psychosomatics* 12 371–379. 5172928 10.1016/S0033-3182(71)71479-0

